# Mutation Accumulation in an Asexual Relative of *Arabidopsis*

**DOI:** 10.1371/journal.pgen.1006550

**Published:** 2017-01-09

**Authors:** John T. Lovell, Robert J. Williamson, Stephen I. Wright, John K. McKay, Timothy F. Sharbel

**Affiliations:** 1 Department of Integrative Biology, University of Texas at Austin, Austin, TX, United States of America; 2 Department of Ecology and Evolutionary Biology, University of Toronto, Toronto, Ontario, Canada; 3 Department of BioAgricultural Sciences and Pest Management, Colorado State University, Fort Collins, CO, United States of America; 4 Apomixis Research Group, Leibniz Institute for Plant Genetics and Crop Plant Research, Gatersleben, Germany; John Innes Centre, UNITED KINGDOM

## Abstract

Asexual populations experience weaker responses to natural selection, which causes deleterious mutations to accumulate over time. Additionally, stochastic loss of individuals free of deleterious mutations can lead to an irreversible increase in mutational load in asexuals (the “click” in Muller’s Ratchet). Here we report on the genomic divergence and distribution of mutations across eight sympatric pairs of sexual and apomictic (asexual) *Boechera* (Brassicaceae) genotypes. We show that apomicts harbor a greater number of derived mutations than sympatric sexual genotypes. Furthermore, in phylogenetically constrained sites that are subject to contemporary purifying selection, the ancestral, conserved allele is more likely to be retained in sexuals than apomicts. These results indicate that apomictic lineages accumulate mutations at otherwise conserved sites more often than sexuals, and support the conclusion that deleterious mutation accumulation can be a powerful force in the evolution of asexual higher plants.

## Author Summary

Most plants and animals reproduce through sex, a form of reproduction associated with many costs (e.g. finding and attracting mates). Asexual reproduction can circumvent these costs; however, it has only evolved in a handful of plant and animal species. Here, we address this evolutionary conundrum, and ask: why does sexual reproduction dominate among multicellular organisms? We find that asexual individuals harbor many more DNA mutations than sexual individuals. Importantly, asexual genotypes are more likely to retain mutations in highly-conserved sites (shared across species) that cause protein changes. Such mutations may be detrimental to asexually reproducing populations, and may increase the likelihood of extinction among lineages that do not undergo sex.

## Introduction

Within finite populations, sexual recombination can improve the probability of fixing beneficial mutations (“Hill-Robertson” effect) [1], while asexual populations are more likely to accumulate deleterious mutations [2–5]. Furthermore, since asexual populations do not undergo recombination, ancestral adaptive genotypes cannot be recovered once deleterious alleles reach fixation, a process known as “Muller’s Ratchet” [6–8]. Combined, these processes are thought to increase the rate of deleterious mutation accumulation in asexual lineages [9], which may favor sexual reproduction. The observation that most obligately asexual plant and animal taxa have evolved very recently [3; but see 10] provides credence to the hypothesis that deleterious mutation accumulation through Hill-Robertson interference and Muller’s Ratchet contributes to the extinction of lineages that never undergo sex.

Like parthenogenetic animals, plants that reproduce asexually via seed (hereon “apomixis”) are commonly hybrid and/or polyploid [11–13], which may buffer the effects of accelerated deleterious mutation accumulation. In addition, if polyploidy is confounded with apomixis, genomic comparisons between sexual and asexual genotypes are difficult to interpret. The genus *Boechera* (Brassicaceae) offers an exceptional case among plants, where apomictic diploids are phylogenetically dispersed across as many as 30% of the >100 species [14–17].

Hybridization is thought to play a role in the spread and maintenance of apomixis in *Boechera*, [14–15, 17–18] despite its potential evolutionary costs. While most apomictic *Boechera* exclusively produce seeds with meiotically unreduced and parthenogenically developed embryos [19–20], some genotypes produce fertile reduced pollen [15,18]. New diploid apomictic lineages may be formed when sexual maternal plants are fertilized by reduced pollen from apomictic genotypes, which may provide a vector to horizontally transmit genetic factors that cause apomixis. While rare, this process could account for the ubiquity of diploid apomictic hybrids across the genus. Additionally, apomixis appears to be an ancient characteristic of *Boechera*, as nearly all apomictic *Boechera* analyzed to date share several identical-by-descent alleles that are not found in sexuals [15,20]. Hence, sufficient evolutionary time may have elapsed to detect deleterious mutation accumulation in apomictic *Boechera*.

Diploid apomictic and diploid sexual genotypes occur in sympatry across many *Boechera* species [15], including *B*. *spatifolia* [16], enabling evolutionary comparisons between mating systems without the confounding effects of ploidy or natural history variation. This unique biology, common self-pollination in sexual genotypes, and recent common ancestry with the model plant *Arabidopsis* together make *Boechera* a premier system to study the evolution of alternative reproductive strategies in plants [14]. Here, we exploit the genetic resources of *Boechera* to test how the process of mutation accumulation differs between sexual and apomictic genotypes. Specifically, we hypothesize that the combined effects of Hill-Robertson interference and Muller’s Ratchet will lead to the accumulation of more deleterious mutations in apomictic compared to sexual genomes.

## Results and Discussion

### Apomicts are characterized by elevated sequence diversity and heterozygosity

**Fig 1:**
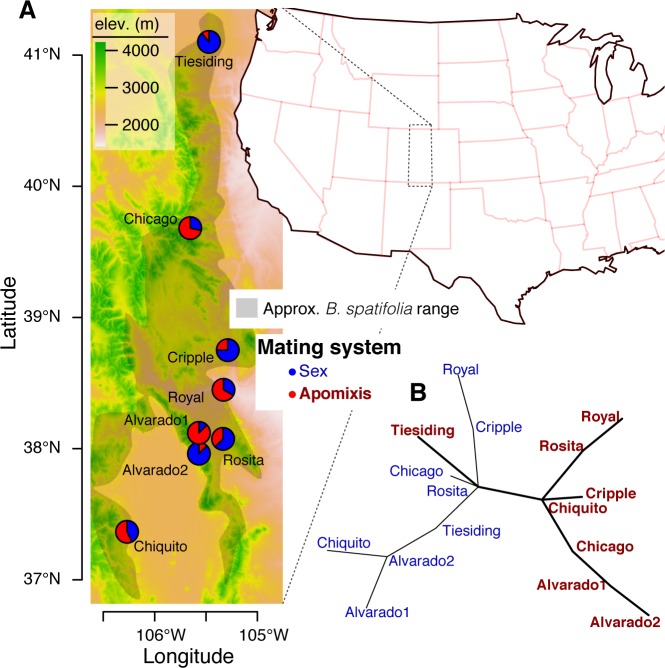
Geographic and genetic structure of mating system and ploidy variation in *B*. *spatifolia*. The geographic locations of sympatric sample populations (**A**) are plotted, where the proportions of sexual and apomictic individuals screened are indicated by blue and red pie chart segments respectively. One individual of each mating system was chosen from each population and sequenced. SNP data from the re-sequenced individuals were used to generate a minimum spanning network where edge length is proportional to genetic distance (**B**). Nodes representing the sexual and apomictic individual are labeled by their population ID and colored according to their mating system.

We compared the genomic DNA sequences of one apomict and one sexual genotype from eight “sympatric” populations where diploids of both mating systems co-occurred (S1 Table, Fig 1A). From the 16 re-sequenced genotypes (8 apomict-sexual sympatric pairs), we called 8.4M high quality single nucleotide polymorphisms (SNPs). Genetic structure analyses indicated that the sexuals and 7/8 apomicts formed two distinct genetic clusters, while the most northern, “Tiesiding” apomict constituted its own group (Fig 1B).

**Fig 2:**
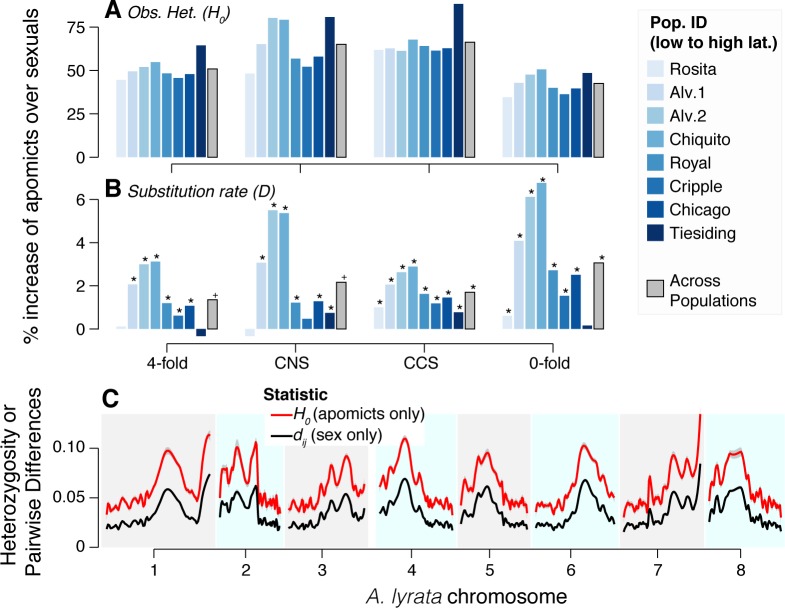
Genomic distribution of SNP diversity. Observed heterozygosity (*H*_*0*_, **A**) and substitution rate (*D*, **B**) were calculated for all populations across four SNP annotation categories. For each apomictic-sexual sympatric pair, the relative increase of the sympatric apomict to the sexual is plotted (colored by population, where the lightest populations are from the south and darkest are northern). Significance of the comparison is presented (Fisher’s test *P* ≤ 0.05*, *P* ≤ 0.1^+^). All comparisons are significant for *H*_*0*_. **C** *H*_*0*_ of each apomictic genotype and proportion of pairwise differences between each sexual genotype (*d*_*ij*_) were calculated for non-overlapping 20k SNP windows. Loess smoothed curves are plotted across these windows.

To understand the degree of DNA sequence divergence within and between mating systems, we calculated observed heterozygosity (*H*_*0*_ = the fraction of polymorphic sites that are heterozygous) and substitution rate (*D* = the fraction of alleles that are different from the *A*. *lyrata* reference). Sexual *Boechera spatifolia* are self-compatible and highly inbred (*F*_*IS*_ = 0.75, ~85% selfing, [21]), while apomicts are typically hybrids. Therefore, it is not surprising that across all sites and populations, apomicts displayed much greater observed heterozygosity (*H*_*0*_ = 0.23, Fig 2A) than sexuals (*H*_*0*_ = 0.15). Apomicts also exhibited a slightly higher substitution rate (*D* = 0.076, Fig 2B) than sexuals (*D* = 0.075, S1 Fig).

The three most southwestern populations (Alvarado 1–2, Chiquito) contained apomicts that were more diverged from the sympatric sexuals than other populations. Distance-based networks defined strong connections between the most southern “Chiquito” apomict and those in the north. Such relatedness between northern and southern populations—but not among the northern populations—potentiated migration or gene flow from the south. As such, it is possible that the southwestern apomictic lineages are older than their northern counterparts and have had more time to accumulate genetic diversity. Furthermore, these data suggest that each apomictic genotype is not the result of a local, recent hybridization. Instead, due to shared alleles and genetic structure among apomicts, it is possible that the seven southern apomicts resulted from less than four distinct hybridization events.

Some asexual taxa are characterized by the introgression of genomic regions (e.g. *Pennisetum/Cenchrus* [22]), or entire chromosomes (e.g. *Daphnia pulex* [23]) from related species, which could account for the observed elevated average *H*_*0*_ of apomicts. If apomicts resulted from isolated introgressions within *B*. *spatifolia*, we would expect to find genomic regions that show significantly elevated *H*_*0*_ among apomicts, but similar densities of pairwise differences (*d*_*ij*_) among sexual genotypes. Instead, we observed strong and consistently elevated apomictic heterozygosity relative to sexual *d*_*ij*_ (Fig 2C), without any low heterozygosity regions in apomicts relative to sexual *d*_*ij*_. Indeed, across the genome, apomictic *H*_*0*_ was >2x higher than sexual *d*_*ij*_ (Fig 2C). Such elevated heterozygosity may be due to the accumulation of mutations in the non-recombining apomictic genomes, which necessarily remained in a heterozygous state (in the absence of gene conversion [24]). These data were also consistent with the hypothesis that the apomicts were hybrids derived from crosses between genotypes that were significantly more diverged than any of the sexual accessions sampled here.

### Hybridization underlies the origin of apomictic lineages

Given these results suggesting elevated heterozygosity, combined with previous results from the *Boechera* genus [18,25], we hypothesized that apomicts would be hybrids, where *B*. *spatifolia* represented one of the parental genotypes. It is possible to leverage the non-recombinant nature of apomictic genomes to test for a hybrid origin. For any given pair of alleles at a single locus, a hybrid apomict would contain a *B*. *spatifolia*–derived haplotype and one derived from another *Boechera* species. Alternatively, if apomixis arose from true breeding sexual *B*. *spatifolia* lineages, both apomictic haplotypes would be equally diverged from the ancestral sexual genome.

**Fig 3:**
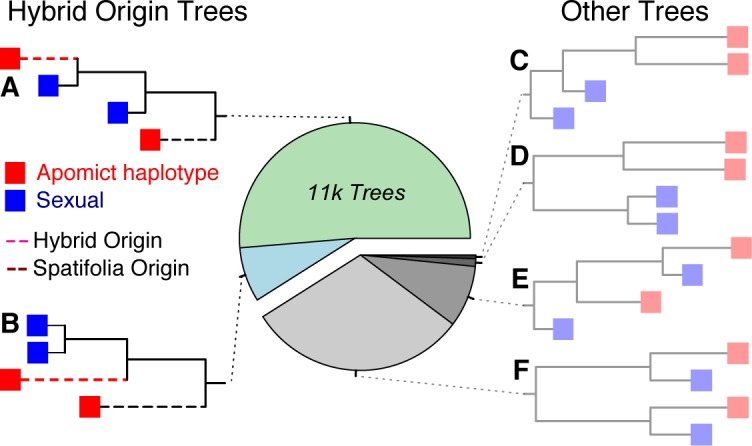
Classification of haplotype trees to infer the impact of hybridization. The ~22k trees generated from strictly filtered 5-sequence haplotype alignments were binned into six possible topologies. The relative abundance of each is plotted (for the distributions of all trees see S3 Fig). Those trees that present a hybrid evolutionary history are plotted on the left (**A**-**B**), while all other threes are on the right (**C**-**F**). Red branch tips indicate the apomictic haplotype, while blue branch tips depict the sexual ‘pseudohaplotype’. In the hybrid trees, the non-*B*. *spatifolia* sub-genome is represented by the most diverged terminal branch (dashed red edge), while the *B*. *spatifolia*-derived sub-genome (dashed black edge) is most closely related to the sexual *B*. *spatifolia* chromosomes. The *A*. *lyrata* rooted edges are not plotted. Branch lengths are averaged across all trees in each bin.

To accomplish a test for hybrid origins of apomicts, we examined the topology of haplotype trees distributed across the genome. For any given apomictic sequence, we performed a phasing analysis [26] whereby alleles at heterozygous loci along overlapping sequencing reads were split and recorded as a pair of phased haploid sequences. Such an analysis was not possible in the recombinant sexual genomes, which have ~50% lower heterozygosity than apomicts (Fig 2A and 2C). Therefore, we captured sexual sequence diversity along the apomictic haplotype regions by creating ‘pseudo-haplotypes’, where alleles at each heterozygous locus were binned randomly into one of the two phases. We then used a maximum likelihood method to construct haplotype trees for each region with the five analyzed sequences: 1–2) two phased apomictic sequences (‘haplotypes’); 3–4) two sexual ‘pseudo-haplotypes’, and 5) the *Arabidopsis lyrata* reference genome. After filtering out trees with low bootstrap support, and trees from regions with statistically elevated heterozygosity among sexual genotypes—which likely correspond with gene duplication events—we were able to analyze >22k trees representing about 15 Mb of the genome (Fig 3).

To test if apomicts were hybrids, or derived solely from sexual *B*. *spatifolia*, we binned the 22k haplotype trees into the six possible topologies. Topologies where one apomictic haplotype was basal while the other was nested within, or sister to, the sexual pseudo haplotypes indicated a hybrid origin (Fig 3A and 3B). Alternatively, a within-species mating would produce a diverged pair of apomict genotypes relative to basal sexual sequences (Fig 3C and 3E). The bulk (59.0%) of the strictly filtered trees had a topology indicative of a hybrid origin (Fig 3A and 3B). The trees that indicate a purely *B*. *spatifolia* origin were much rarer, representing only 10.2% of all topologies. The remaining 30% of alignments fell into tree topologies that were ambiguous—the most common ambiguous tree represented sequences with massive divergence between the two sexual genomes, indicative of duplication. Combined, increased apomictic heterozygosity and the observation that 5/6 unambiguous trees were of hybrid origin, provide strong evidence that *B*. *spatifolia* apomicts were originally derived from hybridization.

Finally, we corroborated the hybrid origin of the sampled apomicts with an analysis of species-specific alleles at 15 microsatellite loci [17] (*Supplementary Note 1*), where alleles of heterozygous genotypes were categorized as *B*. *spatifolia*-specific or derived from another *Boechera* species [27]. We determined that *B*. *fenderli*, a closely allied species to *B*. *spatifolia*, was the most likely hybridizing species in 7/8 of the apomicts; however, the northern-most ‘Tiesiding’ population appeared to be a putative hybrid between *B*. *spatifolia* and *B*. *pendulocarpa* (*Supplementary Note 1*). Combined these analyses demonstrated at least two distinct hybrid origins of apomictic *B*. *spatifolia*.

### Apomicts harbor more deleterious mutations than sexuals

Since sexual *B*. *spatifolia* are heavily inbred, sexual populations have likely purged many deleterious recessive alleles. However, deleterious mutations may persist in apomictic lineages, which display higher heterozygosity and should have weaker responses to selection. To test whether apomicts have accumulated more deleterious mutations than sexuals, we annotated the SNP datasets using the *Arabidopsis lyrata* reference and categorized SNPs based on their location in genes and phylogenetically conserved regions of DNA. Such conserved sites were identified by Haudry et al. (2013) [28] by aligning reference sequences in diverse species in the Brassicaceae and locating regions that were conserved across deep evolutionary time–both coding and non-coding regions were identified this way. At each polymorphic site, the allele that was not present in the *A*. *lyrata* reference genome was determined to be derived. To ensure that we were calling the true ancestral state, we also used Haudry et al. (2013)’s consensus sequence (across the Brassicaceae) to define the derived alleles. Since these two analyses produced very similar results (S2 Fig), we opted to present comparisons with *A*. *lyrata*. In total, we categorized 1.2M SNPs in coding sequences and conserved non-coding sites into four groups: 1) conserved non-coding sites (“CNS”), 2) conserved coding sites (“CCS”), 3) “0-fold” sites, where any mutation causes an amino acid substitution, and 4) “4-fold” degenerate sites, where any mutation is synonymous.

The hypotheses of Muller’s Ratchet and Hill-Robertson interference predict that mutations in conserved sites are more likely to be retained in apomictic than sexual lineages. In order to quantify the strength of this effect we calculated the ratio of mutations at phylogenetically constrained sites to those among unconstrained sites: *d*constrained/*d*neutral, where 4-fold degenerate sites served as the neutral denominator. This statistic is analogous to non-synonymous/synonymous substitution ratios (*dn/ds*), but accounts for synonymous sites that are phylogenetically constrained (e.g. regulatory factors). We expected *d*constrained/*d*neutral values of apomicts to exceed those of sexuals due to mutation accumulation via Muller’s Ratchet and reduced selective elimination of deleterious mutations via Hill-Robertson interference.

**Fig 4:**
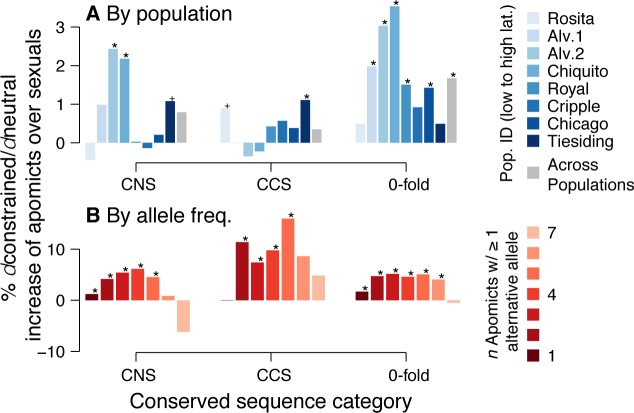
Signatures of relaxed purifying selection in apomicts. We calculated *d*constrained/*d*neutral ratios for each population with bar colors following Fig 2A and 2B (**A**). Additionally, *d*constrained/*d*neutral ratios were calculated from SNPs binned by their derived allele frequency among the 7 southern apomictic genotypes (**B**). For example *n* = 1 indicates a site where a single apomictic genotype has a derived allele, while *n* = 7 represents sites where all apomicts share a derived allele. Significance categories follow Fig 2A and 2B.

Indeed, apomictic genotypes harbored a higher proportion of derived alleles at constrained sites (excess at 0-fold: 3.1%, CNS: 2.1%, CCS: 1.7%) and to a lesser extent at neutral sites (4-fold: 1.3%) compared to sexuals (Fig 2B). This prevalence of mutations in otherwise conserved sites was reflected in highly elevated *d*constrained/*d*neutral ratios in apomicts over sexuals in 0-fold (odds *Z* = 6.0, *P* < 0.0001) and CNS (odds *Z* = 2.7, *P* = 0.003) sites and marginally elevated ratios among CCS sites (odds Z = 1.56, *P* = 0.059; Fig 4A, S1 Fig). The weakest effect involving apomixis was found at conserved coding sites (CCS). Since these sites are subject to the strongest purifying selection, it is possible that CCS loci are less likely to include large numbers of slightly deleterious mutations that will accumulate first following a loss of sex.

It is possible that the genome-wide evidence of increased deleterious mutation accumulation in apomicts (Fig 4A, *Supplementary Note #2*) was due to different alleles having been fixed by selection in the two ancestors of the apomictic hybrids. To differentiate between contemporary mutation accumulation and hybridization, SNPs were coded by the derived allele frequencies among apomicts, where shared sites were likely the result of hybridization or common ancestry (Fig 4B; *n* = 7). The Tiesiding population, which has a different hybridizing parent than the other apomicts, was excluded from this analysis (see *Supplementary Note #1–2*). Analyses controlling for allele frequencies in the apomicts demonstrated nearly identical *d*constrained/*d*neutral ratios between the apomictic and sexual lineages among sites where all apomicts shared derived alleles (Fig 4B; *n* = 7). Therefore, SNPs likely derived from hybridization exhibited similar patterns of mutation accumulation between apomicts and sexuals. Alternatively, the strongest evidence for increased deleterious mutation accumulation among apomicts came from sites with intermediate frequencies of derived alleles (Fig 4B; n = 3–6). Indeed, sites where at least three apomictic genotypes contained a derived allele always displayed significantly elevated *d*constrained/*d*neutral ratios across all phylogenetically constrained sites. These intermediate SNPs may have accumulated in ancestral apomictic lineages and spread vertically to its extant progenitors—a scenario that is conceivable given strong shared ancestry within three clusters of apomictic genotypes (Fig 1B).

To further untangle contemporary mutation accumulation from divergence in the parental genomes, we used our phased sequences to infer the number of mutations that have occurred within apomictic and sexual lineages by comparing the terminal branch lengths [23] of the hybrid trees. Since sexual sequences were not true haplotypes, a conservative null hypothesis for equal mutation accumulation would be 2x longer terminal branches in the apomictic than sexual tips. Our results strongly reject this hypothesis—apomictic branch tips were 2.3x longer than sexuals across all trees (with a relaxed heterozygosity threshold), and 2.7x longer among hybrid origin trees (binomial test *P* < 0.0001 for both tests). As such, mutation accumulation is accelerated in asexuals, regardless of a hybrid origin.

### Increased mutation accumulation in the apomicts is not only due to the hybrid origin but also has occurred since the shift in mating system

Given a hybrid origin of the sequenced apomicts, it is possible to leverage the non-recombinant and hybrid nature of apomictic genomes to test for the presence of Muller’s Ratchet dynamics or Hill-Robertson interference. Thus far, we have found strong evidence for elevated mutation density across all sites in apomictic relative to sexual genomes. However, such composite estimates of mutation accumulation cannot conclusively separate the effects of hybridization and contemporary mutations. To definitively test for differences in contemporary mutation accumulation, we exploited the fact that these apomictic *Boechera* genomes were non-recombinant and of hybrid origin. Hence, each apomictic genotype contained a haploid genome derived from a sexual *B*. *spatifolia*, and one from a sister species. A previously sexual genome (haplotype) should begin to accumulate mutations in a Muller’s Ratchet-like process upon its introduction into a newly formed hybrid apomictic lineage. By comparing sequence divergence between the sexual *B*. *spatifolia* pseudo-haplotypes and the *B*. *spatifolia*-derived apomictic haplotype, we were able to determine the number and type of mutations that have accumulated within extant apomictic lineages. These analyses were constrained to the terminal branches of alignments in coding regions, as these are most likely to represent single copy loci with uniquely mapping reads in trees with topologies indicative of hybridization (Fig 3). It is important to note that such polymorphism analysis on haplotype trees represented a very conservative estimate of mutation accumulation. Mutations in extant apomictic lineages clearly accumulated on both homologous sequences; however, since it is impossible to separate contemporary mutations from those that are ancestral in the hybridizing genotype—without having genomic sequence information from the hybridizing conspecific—we must exclude such sites to definitively infer the effects of relaxed purifying selection.

**Fig 5:**
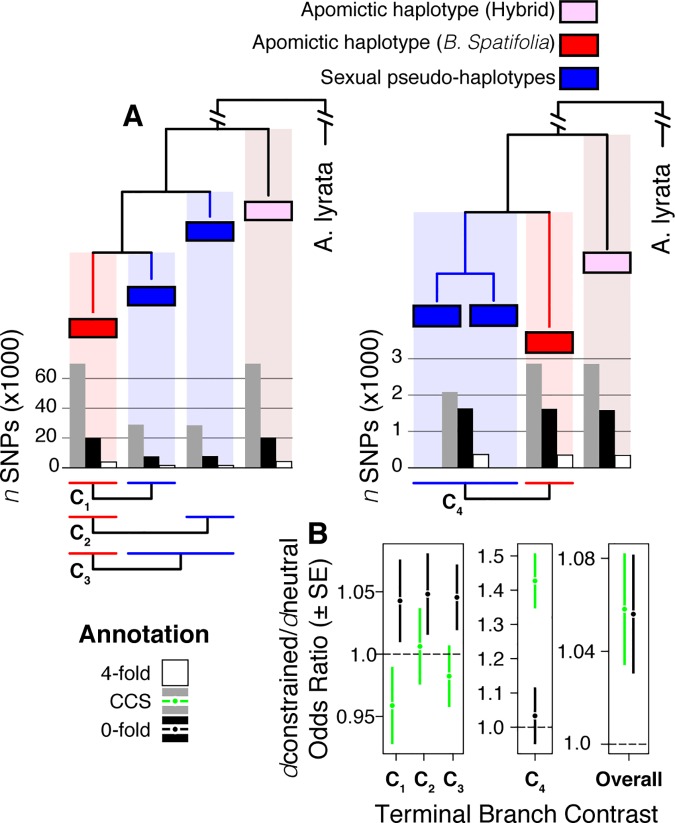
Classification of mutation accumulation within haplotype trees. We documented the types and number of mutations on the terminal branches of the two hybrid trees (Fig 3A and 3B). For both trees, the total numbers of coding SNPs along the terminal branches are plotted below the branch tips as vertical bars (**A**). The discrepancy in SNP numbers between trees is due to the presence of many more haplotypes belonging to the first (Fig 4A) topology. The sexual terminal branches were compared to the *B*. *spatifolia*-derived haplotype terminal branch directly. For the left tree, this involved three contrasts; however, since the sexual pseudo-haplotypes cluster together in the right tree, it was appropriate to group these and make only a single contrast. Fisher’s test odds ratios (± odds ratio SE) of the contrasts (labeled C_1_ –C_4_) are expanded as well as the overall Fisher’s test across all contrasts (**B**).

*B*. *spatifolia* haplotypes from apomictic lineages contained more mutations at conserved sites than haplotypes from sexual *B*. *spatifolia* lineages (Fig 5A). Indeed, there were ~37k (23%) more derived mutations in apomictic than sexual terminal branches, demonstrating greater mutation accumulation within *B*. *spatifolia*-derived apomictic chromosomes. Furthermore, the ratio of conserved to neutral site mutations was significantly greater in apomict than sexual haplotypes. Across all hybrid trees, the apomictic terminal branch was associated with a higher odds ratio of 0-fold (Fisher’s odds = 1.056, *P* = 0.017) and CCS mutation rates (odds = 1.058, *P* = 0.0096) relative to neutral 4-fold sites (Fig 5B), indicating that apomictic genomes were more likely to accumulate deleterious mutations than sexual genomes.

### Conclusions

Recombination that accompanies sex increases the potential for adaptation relative to non-recombinant lineages. Conversely, deleterious mutations are fixed at an accelerated rate in asexual populations. Combined, our data demonstrated two levels of genome sequence divergence between *Boechera* mating systems. As expected, divergence was driven primarily by hybridization, as this is the mechanism through which apomixis spreads. However, contemporary mutation accumulation also impacted the molecular evolution of apomicts. In particular, these results demonstrated that apomicts were accumulating mutations in otherwise conserved sites, a pattern consistent with the predictions of Hill-Robertson interference and Muller’s Ratchet.

## Methods

### Plant collection and growth

In 2012–2013, seeds were collected from >5 fruits of >8 maternal plants per population. Plants analyzed here were grown from seeds generated from self-pollinated maternal plants (localities and collection information have been published previously [16]). Seeds from each of 16 genotypes were germinated directly in 1” diameter RLC-4 containers (Steuwe and Sons, Tangent, OR, USA) filled with Fafard 4P soil mix. Three seeds were placed on the soil and germinated following 14 days of cold stratification. Seedlings were subsequently thinned to a single plant. Details regarding growth conditions were published previously [16]. In short, plants were grown in a single Conviron ATC60 growth chamber at Colorado State University, Ft. Collins, CO, USA programmed as follows: days 1–14: 23/18°C, 12/12h day/night; days 15–21; 18/8°C, 12/12h day/night; days 22–54: 8/4°C, 8/16h day/night; days 54-: 23/18°C, 12/12h day/night.

Mating system was determined by screening seed ploidy of both the wild-collected maternal plants and self-pollinated, greenhouse-grown plants using the Flow Cytometric Seed Screen (FCSS) using a Partec PAII flow cytometer at the Institute for Plant Genetics and Crop Plant Improvement (IPK), Gatersleben, Germany. In diploid sexual *Boechera*, self pollinated seed will have triploid (3n) endosperm and 2n embryo cells. However, diploid apomictic *Boechera* ovules have unreduced polar and egg nuclei. Apomictic eggs develop parthenogenically, but the polar nuclei are fertilized by a single pollen cell. Therefore, apomictic seeds retain 2:5 or 2:6 ploidy ratios of the developed embryo and endosperm (depending on pollen ploidy). The FCSS permits identification of these ratios, reliably determining the mating system of the maternal plant. All genotypes were screened using three separate 5-seed bulks [16]. Additionally, 96 individual seeds were screened from the genotypes subjected to HiSeq resequencing following the methods of Aliyu et al. (2011) [19].

### DNA sequencing

The three most recently expanded leaves from mature plants were harvested, placed onto ice and immediately freeze-dried. Genomic DNA was extracted using the Qiagen DNEasy Plant Miniprep kit (Qiagen Corp. Germantown, MD, USA) following manufacturer protocols (qiagen.com). DNAs were extracted from a set of 16 genotypes and subjected to 2x150 PE sequencing on the Illumina platform at the GenomeCanada facility at McGill University, Montreal, Canada.

### SNP analysis

All bioinformatics scripts have been annotated and are published on github (github.com/williarj/Boechera_Mutation_Accumulation). Reads were aligned to the *A*. *lyrata* reference genome using *bwa* [29] and *stampy* [30] with default parameters. We also explored alignments to the *Capsella rubella* genome, which diverged from the *Boechera* lineages ~14.7 MYA [14]. However, alignment statistics indicated that *A*. *lyrata* would serve as a better reference

The *GATK* v2.7–4 UnifiedGenotyper [31] was used to call both SNPs and insertion-deletion polymorphisms for each sample after removing duplicates and insertion-deletion realignment. Sites with a quality score below 15 and individual genotypes with a quality score below 40 were removed from the analysis. In order to remove error prone (e.g. repetitive) regions, the genome was split into 20kb windows; those where less than 30% of sites passed all other filters were removed from further analyses. The number of NA calls was consistent across samples (standard deviation of %NA = 0.47%). Finally, we retained only SNPs that were polymorphic among our samples. SNPs were annotated via SNPeff [32] against the *A*. *lyrata* reference annotation [33]. CNS and CCS SNPs were annotated from conserved coding and non-coding sequences defined by Haudry et al. (2013) [28], who used the degree of phylogenetic constraint across the Brassicaceae as a measure of historical purifying selection.

It is important to note that *Boechera* and *Arabidopsis* are significantly diverged. Such DNA sequence divergence may pose a problem if there was a paucity of called sites in moderately conserved regions. Overall, we called 62.1Mbp (30.0%) of the *A*. *lyrata* genome. The bulk of the uncalled regions resided in non-coding, non CNS or pericentromeric regions (S4 Fig). However, we were able to make 26.5M coding and 3.8M CNS calls, which represented 75.3% and 83.8% of the total coding and conserved non-coding sequences in the *A*. *lyrata* genome. Such high coverage of the sites used for statistical comparisons in the analyses presented here decreases the likelihood of systematic bias in our data.

Counts of SNP alleles for each annotation category were generated for each of the 16 genotypes and the total number of sites with allele calls and number of polymorphic sites were counted. Using the number of alternative homozygous genotypes and heterozygous genotypes, we were able to calculate two statistics: observed heterozygosity (*H*_*0*_ = *n* Heterozygotes / *n* polymorphic sites) and substitution rate (*D* = ((.5 * *n* Heterozygotes) + *n* Alternative Homozygotes) / *n* sites with genotype calls). We also calculated the ratio of substitutions at conserved and neutral sites as D (0-fold, CNC, CNS sites) / D (4-fold sites). We calculated the statistical difference between mating systems via Fisher’s tests (*H*_*0*_ and *D*) or Z-tests of odds-ratios differences (*d*constrained/*d*neutral ratios). Both tests were accomplished with R base functions.

### Haplotype analysis

HAPCUT v0.7 [26] was used to generate haplotypes in all apomictic samples using a max insert size of 600. For each pair of HAPCUT-split apomictic haplotypes, a three-sequence alignment was built, including the *A*. *lyrata* reference and the two apomictic haplotype sequences. Sexuals, which have significantly lower heterozygosity (Fig 2), were less likely to generate long enough sets of overlapping heterozygous reads. As such, culling site to those with long enough haplotypes for both sexual and apomictic genotypes biased our analysis to those sites that were highly heterozygous in the sexual genotypes. Such sites may be the result of inefficient or erroneous mapping. To overcome this confounding factor, we chose to generate “pseudo-haplotypes”, rather than use HAPCUT-based haplotype splitting for the sexual genotypes. We generated pseudo-haplotypes by randomly assigning the alleles at each heterozygous site to one of two fasta files. The resultant pair of pseudo-haplotype fastas were concatenated with the 3-sequence alignments generated by HAPCUT. Since the sexual lineages recombine these pseudo-haplotypes should represent real possible haplotypes within these populations.

Maximum likelihood phylogenetic trees (with bootstrap values) were generated from the 5-sequence alignments using *RAxML* v8.1.17 [34] with the GTRGAMMA nucleotide substitution model. Once trees were generated, we counted the number of derived alleles on each branch of each tree and quantified the number of trees matching each possible tree topology. Mutations on each branch were binned into one of five categories: CNS, CCS, 0-fold, 4-fold, other.

We assumed that the apomictic haplotype that most closely resembles sexual pseudo-haplotypes was derived from the sexual *B*. *spatifolia* pedigree. In this case, the node distinguishing this apomictic haplotype (see Figs 2 and 4) and the most closely related sexual pseudo-haplotype represents a conservative estimate of the coalescence of these two lineages. We calculated mutation accumulation of the extant apomictic lineage as the number of derived alleles assigned to the terminal branch of the *B*. *spatifolia*-derived apomictic haplotype. To document the differential rate of mutation accumulation between sexual and apomictic lineages, we applied the statistics described above to the number of mutations assigned to the number of mutations on terminal branches representing the *B*. *spatifolia*-derived apomictic haplotype and the two sexual pseudo-haplotypes.

## Supporting Information

S1 Text Two notes are presented herein: Microsatellite analysis to determine parental origins of apomictic hybrids (*note 1*). Analysis of polymorphism controlling for apomictic allele frequency (*note 2*).
(PDF)

S1 Table The geographic locations and sample sizes of all *B*. *spatifolia* populations sampled. The populations subjected to whole-genome resequencing are in bold.
(PDF)

S1 Fig Observed heterozygosity, dconstrained/dneutral and substitution rate of each sample. These data were used to make the calculations presented in Fig 2A and 2B and Fig 4.
(PDF)

S2 Fig Evidence for elevated mutation accumulation in asexuals does not depend on the reference sequence. Population genetic comparisons were calculated following the methods for Fig 2A and 2B and Fig 4A, but by using the Brassicaceae consensus reference sequence (Haudry et al. (2013). These results largely recapitulate those presented in the main text, which use *A*. *lyrata* as the reference sequence.
(PDF)

S3 Fig Distribution of haplotype trees prior to filtering by heterozygosity and bootstrap support. This analysis matches that of Fig 3, but with all haplotypes that passed length filtering.
(PDF)

S4 Fig Mapping efficiency and coverage summary of alignments to the *A*. *lyrata* reference genome. Here, we present the fraction of sites in the *A*. *lyrata* reference genome called in our analysis. This serves as a summary of the performance of mapping to a divergent reference sequence. The physical position is presented on the x-axis with the same scale for each chromosome. Note that the proportion of called sites is low in pericentromeric regions, but high in the chromosome arms.
(PDF)
