# Supplementary material for: Mutation Accumulation in an Asexual Relative of Arabidopsis
Source: PLoS Genet. 2017 Jan 9;13(1):e1006550. doi: 10.1371/journal.pgen.1006550 (PMC5261742; doi:10.1371/journal.pgen.1006550)
Supplement: S1 Text — Analysis of polymorphism controlling for apomictic allele frequency (note 2). (PDF) [file pgen.1006550.s001.pdf]

Online Supplementary material for: Lovell et al. *Mutation accumulation in an asexual relative of Arabidopsis*.

SUPPLEMENTARY NOTES

Supplementary Note 1: Microsatellite analysis to determine parental origins of apomictic hybrids. The three most recently expanded leaves from mature plants were harvested, placed onto ice and freeze-dried. Genomic DNA was extracted using the Qiagen DNEasy Plant Miniprep kit (Qiagen Corp. Germantown, MD, USA) following manufacturer protocols (qiagen.com). Aliquots of the DNAs were subjected to genetic analysis of microsatellites. The 15 SSR markers were genotyped following previously published protocols [4-6] and used to compare allelic states with previously genotyped *Boechera* lines (M.D. Windham[6,7]). Since true phylogenetic analyses are not appropriate for the assessment of hybrids, we used the *Boechera Microsatellite Website* database to infer parentage. The program PRIUS (Li et al. in review [8]) infers putative parentage based on SSR similarity across > 4k *Boechera* specimens (http://sites.biology.duke.edu/windhamlab/). The inferred parentage presented in our results are the top hits for each sample submitted.

Supplementary Note 2: Analysis of polymorphism controlling for apomictic allele frequency. In order to quantify selective effects on new mutations that have occurred since the hybridization event leading to apomixes we calculated statistics for sites binned by derived allele frequency in the apomicts. It is important to note that we found strong evidence that the lone northern population "Tiesiding", was a *B. spatifolia x B. pendulocarpa* hybrid while all other apomicts were *B. spatifolia x fenderli* (Fig. S3). Tiesiding was excluded from all subsequent genome-wide SNP analyses because any alleles derived from *B. pendulocarpa* would appear as private among our SNPs. After excluding the northern "Tiesiding" population, the number of reference alleles was counted for each of the seven possible apomictic genotype frequencies. It is not possible to count the total number of sites with allele calls for each allele frequency group (since these sites are necessarily qualified as only those with polymorphism). Therefore, to maintain consistency with previous analyses, the denominator in calculations of D was the total number of sites with allele calls across the entire genome * the proportion of SNPs binned into each allele frequency grouping.

Shared sites were likely the result of hybridization or common ancestry (n = 7, where all apomictic genotypes shared a derived allele, Fig. 3), whereas singleton loci likely evolved within the sampled apomictic genome (n = 1). For all analyses of hybrid alleles we exclude the Tiesiding samples because it is likely derived from an independent hybridization event (Fig. S3).

SUPPLEMENTARY REFERENCES

1. Elshire RJ, Glaubitz JC, Sun Q, Poland JA, Kawamoto K, Buckler ES, et al. A Robust, Simple Genotyping-by-Sequencing (GBS) Approach for High Diversity Species. Orban L, editor. PLoS ONE. Public Library of Science; 2011;6: e19379. doi:10.1371/journal.pone.0019379

2. GOUDET J. hierfstat, a package for r to compute and test hierarchical F-statistics. Mol Ecol Notes. Blackwell Science Ltd; 2005;5: 184–186. doi:10.1111/j.1471-8286.2004.00828.x

3. Paradis E, Claude J, Strimmer K. APE: Analyses of Phylogenetics and Evolution in R language. Bioinformatics. Oxford University Press; 2004;20: 289–290. doi:10.1093/bioinformatics/btg412

4. Lovell JT, McKay JK. Ecological genetics of range size variation in Boechera spp. (Brassicaceae). Ecology and Evolution. 2015;5: 4962–4975. doi:10.1002/ece3.1746

5. Lovell JT, Grogan K, Sharbel TF, McKay JK. Mating system and environmental variation drive patterns of adaptation in Boechera spatifolia(Brassicaceae). Mol Ecol. 2014;23: 4486–4497. doi:10.1111/mec.12879

6. Beck JB, Alexander PJ, Allphin L, Al-Shehbaz IA, Rushworth C, Bailey CD, et al. DOES HYBRIDIZATION DRIVE THE TRANSITION TO ASEXUALITY IN DIPLOID BOECHERA? Evolution. Blackwell Publishing Inc; 2012;66: 985–995. doi:10.1111/j.1558-5646.2011.01507.x

7. Alexander PJ, Windham MD, Beck JB, Al-Shehbaz IA, Allphin L, Bailey CD. Molecular Phylogenetics and Taxonomy of the Genus <I>Boechera</I> and Related Genera (Brassicaceae: Boechereae). Systematic Botany. 2013;38: 192–209. doi:10.1600/036364413X661917

8. Li, F.-W., C. A. Rushworth, J. B. Beck and M. D. Windham. The Boechera Microsatellite Website: an online portal for species identification and determining hybrid parentage. Database (in review)
