# Supplementary material for: Mutation Accumulation in an Asexual Relative of Arabidopsis
Source: PLoS Genet. 2017 Jan 9;13(1):e1006550. doi: 10.1371/journal.pgen.1006550 (PMC5261742; doi:10.1371/journal.pgen.1006550)
Supplement: S1 Table — The populations subjected to whole-genome resequencing are in bold. (PDF) [file pgen.1006550.s002.pdf]

Population Name	LATITUDE	LONGITUDE	alt (m)	Number of Families	Proportion apomixis	ploidy
103	40.0145	-105.5106	2792	8	0	2
105	40.1692	-105.4739	2596	8	0	2
377	38.9373	-106.1466	2886	8	0	2
**Alvarado1**	38.0775	-105.5637	3031	6	0.167	2
**Alvarado2**	38.0775	-105.5637	3031	8	0.875	2
Antero	39.0317	-105.9862	2804	8	0	2
Barr	38.8551	-104.941	2370	6	0	2
Bellaire	40.7507	-105.6126	2606	4	0	2
Brookvale	39.6306	-105.4464	2636	6	0	2
Central	39.8266	-105.5418	2859	8	0	2
**Chicago**	39.6834	-105.6487	3087	7	0.625	2
**Chiquito**	37.3723	-106.2677	2663	8	0.5	2
Cotopaxi	38.3735	-105.6705	2133	7	0	2
**Cripple**	38.7543	-105.281	2474	8	0.25	2
Crosier	40.4499	-105.4468	2463	7	0	2
Crystal	41.1537	-105.1917	2159	5	0	2
Delnorte	37.6322	-106.3631	2493	7	0	2
Gardner	37.783	-105.1305	2128	6	0	2
Goose	39.1762	-105.3843	2803	8	0	2
Green	38.5108	-106.1842	2722	5	0.75	2
Hondo	37.0234	-106.2138	2805	8	1	2
Pinegrove	39.3526	-105.3745	2414	6	0	2
Poso	37.9081	-106.4276	2904	8	1	3
Prince	38.7135	-106.2216	3294	7	0.125	2
**Rosita**	38.0795	-105.3274	2714	5	0.375	2
Round	38.4015	-106.0626	2706	7	0.125	2
**Royal**	38.4531	-105.3226	1942	2	0.666666667	2
Salida	38.4901	-106.0015	2770	5	0	2
Sanluis	37.1922	-105.4493	2475	8	0	2
**Tiesiding**	41.1001	-105.465	2419	7	0.5	2
Virginiadale	40.9658	-105.3761	2238	5	0	2
