# Supplementary material for: Mutation Accumulation in an Asexual Relative of Arabidopsis
Source: PLoS Genet. 2017 Jan 9;13(1):e1006550. doi: 10.1371/journal.pgen.1006550 (PMC5261742; doi:10.1371/journal.pgen.1006550)
Supplement: S1 Fig — These data were used to make the calculations presented in Fig 2A and 2B and Fig 4. (PDF) [file pgen.1006550.s003.pdf]

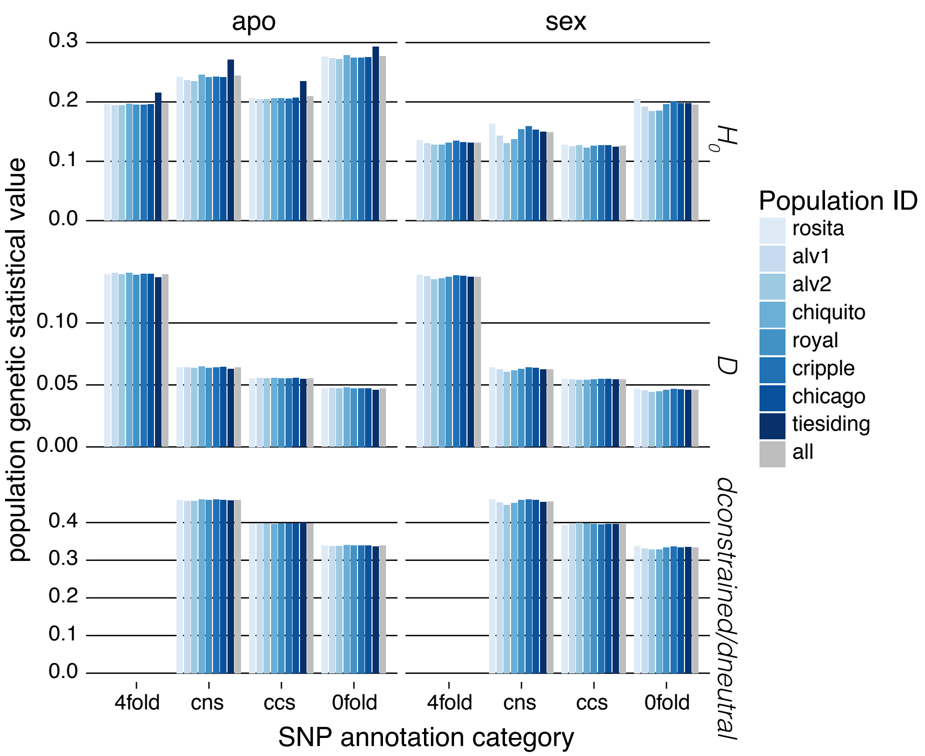
apo
sex
0.3
0.2
0.1
0.0
0.10
0.05
0.00
0.4
0.3
0.2
0.1
0.0
H_0
D
dconstrained/dneutral
population genetic statistical value
4fold
cns
ccs
0fold
4fold
cns
ccs
0fold
SNP annotation category
Population ID
rosita
alv1
alv2
chiquito
royal
cripple
chicago
tiesiding
all
