# Supplementary figures and images for: Mutation Accumulation in an Asexual Relative of Arabidopsis
Source: PLoS Genet. 2017 Jan 9;13(1):e1006550. doi: 10.1371/journal.pgen.1006550 (PMC5261742; doi:10.1371/journal.pgen.1006550)

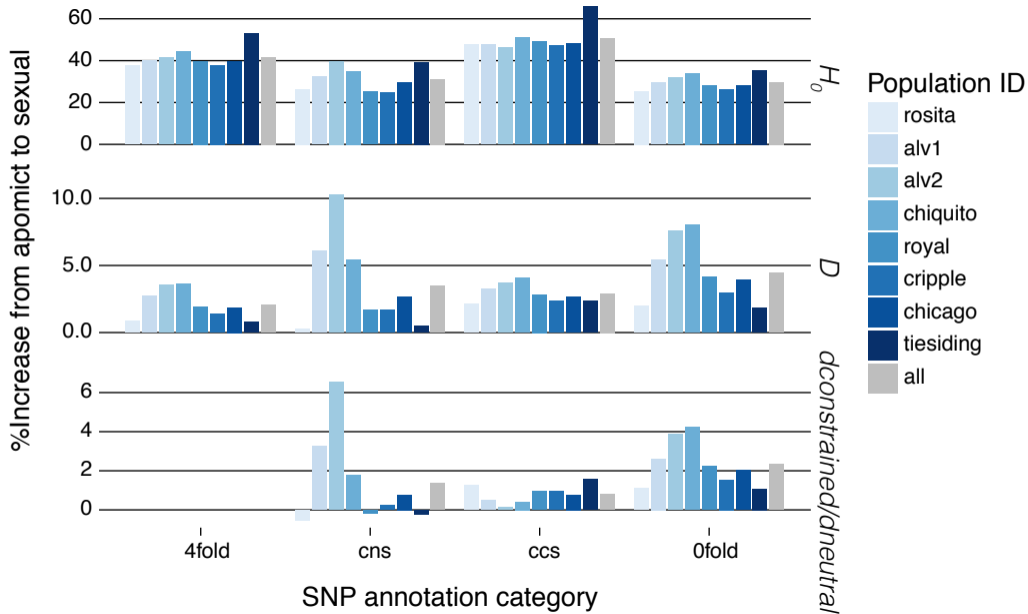

Supplement: S2 Fig — Population genetic comparisons were calculated following the methods for Fig 2A and 2B and Fig 4A, but by using the Brassicaceae consensus reference sequence (Haudry et al. (2013). These results largely recapitulate those presented in the main text, which use A. lyrata as the reference sequence. (PDF) [file pgen.1006550.s004.pdf]

## Hybrid Origin Trees

## Other Trees

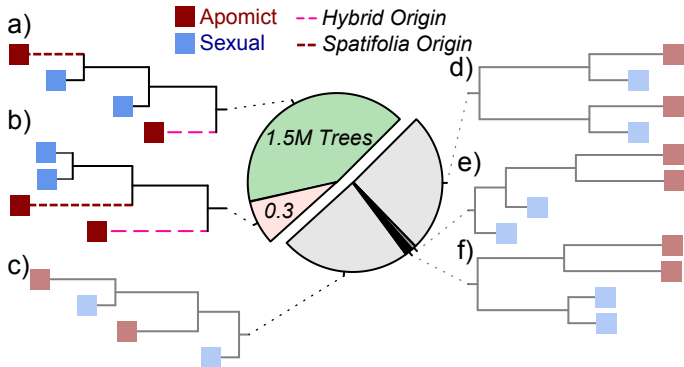

Supplement: S3 Fig — This analysis matches that of Fig 3, but with all haplotypes that passed length filtering. (PDF) [file pgen.1006550.s005.pdf]

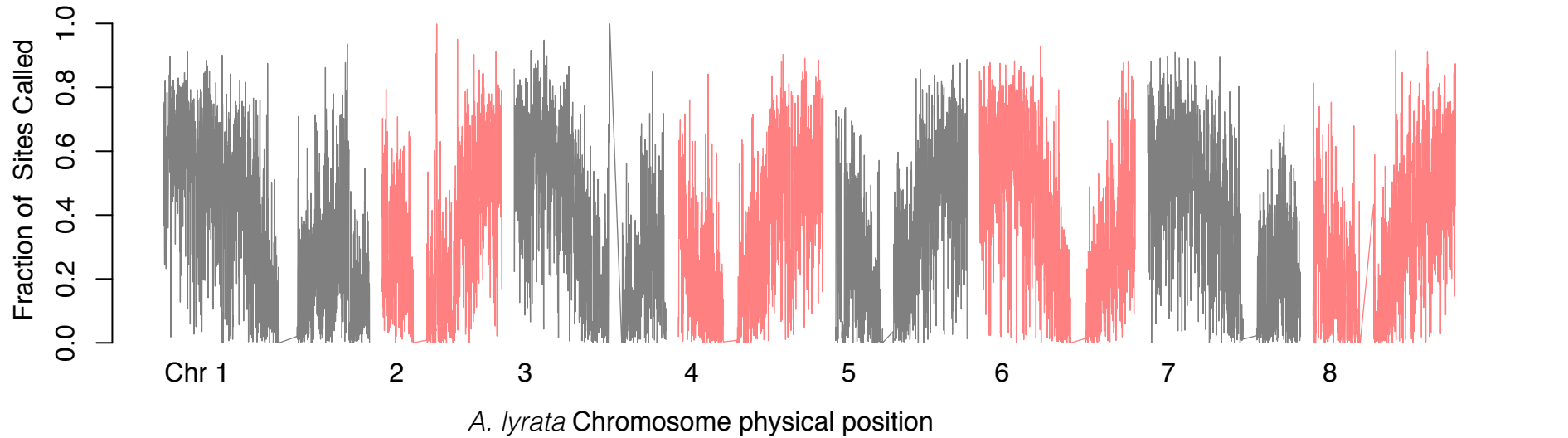

Supplement: S4 Fig — Here, we present the fraction of sites in the A. lyrata reference genome called in our analysis. This serves as a summary of the performance of mapping to a divergent reference sequence. The physical position is presented on the x-axis with the same scale for each chromosome. Note that the proportion of called sites is low in pericentromeric regions, but high in the chromosome arms. (PDF) [file pgen.1006550.s006.pdf]
